# Soil chemical fumigation alters soil phosphorus cycling: effects and potential mechanisms

**DOI:** 10.3389/fpls.2024.1289270

**Published:** 2024-05-24

**Authors:** Yan Wang, Darrell W. S. Tang

**Affiliations:** Soil Physics and Land Management Group, Wageningen University, Wageningen, Netherlands

**Keywords:** soil chemical fumigation, phosphorus solubilizing microorganisms, soil phosphorus cycle, soil phosphorus availability, crop yield, phosphorus fertilization

## Abstract

Soil chemical fumigation is an effective and popular method to increase agricultural productivity. However, the broad-spectrum bioactivity of fumigants causes harm to soil beneficial microorganisms involved in the soil phosphorous cycle, such as soil phosphorus solubilizing microorganisms (PSMs). We review the effects of soil chemical fumigation on soil phosphorus cycling, and the potential underlying mechanisms that ultimately lead to altered phosphorus availability for crops. These complex processes involve the highly diverse PSM community and a plethora of soil phosphorus forms. We discuss phosphatizing amendments aimed at counteracting the possible negative effects of fumigation on phosphorus availability, phosphorus use efficiency, and crop yields. We also emphasize distinguishing between the effects on soil phosphorus cycling caused by the chemical fumigants, and those caused by the fumigation process (e.g. plastic mulching). These are typically conflated in the literature; distinguishing them is critical for identifying appropriate amendments to remediate possible post-fumigation soil phosphorus deficiencies.

## 1 Soil chemical fumigation

Fumigants are broad-spectrum chemical pesticides that once injected into the soil, vaporize and easily diffuse through soil pores, eliminating most soil organisms (Yates et al., 2002; Rokunuzzaman et al., 2016). Many fumigants with various chemical structures, physical properties, and pest elimination mechanisms, such as methyl bromide (MeBr), 1,3-dichloropropene (1,3-D), chloropicrin, metam-sodium, and dazomet, have been developed for use in agriculture (**Table 1**). MeBr previously dominated the agricultural fumigant market, but was phased out due to increased recognition of the damage it causes to the ozone layer (Sun et al., 2018). Other compounds, such as 1,3-D, chloropicrin, metam-sodium, and dazomet, were developed and gained popularity thereafter, as replacements for MeBr (Ruzo, 2006).

**Table 1:** Basic properties of various common chemical fumigants related to their environmental fate, obtained from the Pesticide Properties DataBase (PPDB) of Lewis et al. (2016).

Name	Chloropicrin	1,3-dichloropropene Cis/Trans isomer	Dazomet	Methyl bromide	Metam-sodium	Methyl isothiocyanate
**Chemical formula**	CCl_3_NO_2_	C_3_H_4_Cl_2_	C_5_H_10_N_2_S_2_	CH_3_Br	CH_3_NHCS_2_Na	C_2_H_3_NS
**Solubility in water at 20°C (mg L^-1^)**	2021	2450/2520	3500	13200	578290	8940
**Soil degradation (DT50) (days) (lab at 20°C)**	4.2	10.3	0.52	1	0.01	2.1
**Vapour Pressure at 20°C (mPa)**	4.23**_·_** 10^6^	3.76**_·_** 10^6^/2.98**_·_** 10^6^	1.1	1.90**_·_** 10^8^	57.4	1.74**_·_** 10^6^

All of the substances listed above are classified as highly soluble in water (solubility in water at 20°C > 500 mg L^-1^), mobile (based on sorption behavior), and non-persistent in the soil (DT50 < 30 days). These substances are highly volatile, except dazomet and metam-sodium, which are non-volatile but decompose into volatile substances such as methyl isothiocyanate (Fang et al., 2018). More details of the properties of various common chemical fumigants can be found in Ajwa et al. (2010); Lewis et al. (2016), and Ruzo (2006).

Fumigants effectively control various classes of soil-borne pests and pathogens, and thereby increase crop yields, through several modes of action, including: 1) destroying microbial membrane systems by interfering with the synthesis of lipids, sterols and other components; 2) interfering with the biosynthesis of amino acids and proteins; 3) breaking signal transductions that occur in the cell membranes and hindering the functions of certain proteins; 4) affecting microbial respiration by inhibiting the activities of enzymes such as NADH oxidoreductase, succinate-dehydrogenase and cytochrome bc1; and 5) disrupting microbial nucleic acid synthesis, mitosis and cell division (Ibekwe, 2004; Yang et al., 2011; Castellano-Hinojosa et al., 2022).

In addition to targeting pathogenic organisms, fumigants may due to their broad biocidal activities also influence non-target soil beneficial functional microorganisms, such as the organic material decomposer *Actinobacteria*, and plant disease-controlling fungal genera such as *Mortierella* (Dangi et al., 2015; Zhang et al., 2019b; Castellano-Hinojosa et al., 2022). Variations in soil functional microorganisms may then alter other soil ecosystem functions, including the cycling of various soil nutrients. Chemical fumigation has been shown to affect soil nitrogen, carbon and potassium cycling dynamics, primarily mediated through changes to the soil microorganism community (Castellano-Hinojosa et al., 2022; Sennett et al., 2022, 2023; Sun et al., 2023). However, the mechanisms underlying the effects of chemical fumigation on soil phosphorus (P), have thus far been less researched. P is a key elemental nutrient for plant growth, accounting for nearly 0.2% of plant dry weight (Achary et al., 2017). Li et al. (2017) found that soil chemical fumigation significantly decreased the counts of various microorganisms, some involved in P solubilization processes. Wang et al. (2022a) found that soil chemical fumigation alters soil inorganic P solubilization and soil organic P mineralization processes, and ultimately also soil P availability for uptake by crops. However, the mechanisms linking these findings (i.e. how soil chemical fumigation affects P-related microorganisms, and how this subsequently translates into effects on soil P cycling processes) are currently not well understood, and this forms the main issue addressed in this review. We first draw upon the literature to briefly summarize key concepts of the different soil P forms and their availability for uptake by plants, and show that the effects of soil chemical fumigation on soil P availability are mainly mediated by effects on soil microorganisms. We then expound upon possible mechanisms for these effects by analyzing existing literature, and discuss how future research may address knowledge gaps in our understanding of these mechanisms.

## 2 Soil phosphorus cycling

In plants, P is involved in energy storage and transfer in metabolic pathways, the construction of important cellular constituents, and cell signaling processes (Chiou and Lin, 2011; Veneklaas et al., 2012). Therefore, P is essential for seed germination, flowering and fruiting, promotes root development, and improves the quality of crops (Marschner, 2011; Krishnaraj and Dahale, 2014). P deficiency may be the most important nutritional disorder affecting crop plants (Fageria and Nascente, 2014). Soil P is the sole source of P for uptake by plants, and only dissolved orthophosphate (H_2_PO_4_^-^, HPO_4_^2-^, PO_4_^3-^) can be directly taken up by plants. More than 99% of soil total P cannot be absorbed by plants until they are solubilized or mineralized (Shen et al., 2011; Sharma et al., 2013). Therefore, over 30% of the world’s soil area is P deficient for plant growth due to poor availability rather than insufficient P content (Vance et al., 2003). Phosphate fertilizers are thus essential yet non-renewable resources, which suggests the need to improve soil P availability, and avoid a potential P crisis that may lead to famine and the collapse of agricultural systems (Kopriva and Chu, 2018; Alewell et al., 2020).

Soil P occurs in inorganic P (Pi) and organic P (Po) forms according to their chemical composition and structure (Weihrauch and Opp, 2018). Soil Pi exists in the soil solution as orthophosphates (H_2_PO_4_^-^, HPO_4_^2-^, PO_4_^3-^), and can be bound to soil particles through physisorption and chemisorption processes (Jiang et al., 2015). The transformation of soil inorganic P is highly dependent on soil pH (Hinsinger, 2001; Zhou et al., 2018). In acidic soils, dissolved P is mainly adsorbed to Fe/Al/Mn-oxyhydroxides, clay minerals and metal cations in humic substances through surface electrical charges. As soil pH increases, soil particles become increasingly negatively charged, whereupon bound P is exchanged with OH^-^ through ligand exchange or ligand-promoted dissolution and released freely into the soil solution, improving P availability (Fageria & Barbosa Filho, 2008; Shen et al., 2011; Weihrauch & Opp, 2018). In alkaline soils, the binding of orthophosphate to Ca^2+^ dominates due to the increased replacement of H^+^ by Ca^2+^ on the surfaces of oxyhydroxides, leading to Ca-P-mineral precipitation when P anion concentration becomes high enough in the soil solution. Precipitated Ca-P-minerals can also dissolve and become available by reacting with carbonic acid when the pH values decrease (Shen et al., 2011; Weihrauch and Opp, 2018). Hence, Pi is most soluble and available in soil solution within the narrow pH range of 6.0~6.5 (Abolfazli et al., 2012).

Organic P (Po) contains carbon-hydrogen bonds and are grouped into phosphate esters, phosphonates and phosphoric acid anhydrides, according to the structure of their phosphorus bonds (Huang et al., 2017): 1) Phosphate esters, including the C-O-P functional group, are classified into phosphate monoesters and diesters according to the number of moieties per phosphorus. Phosphate esters [mainly in the form of phytic acid (*myo*-inositol hexaphosphates)] account for more than 50% of soil organic P, but are sparingly soluble and slowly mineralized because they can either be complexed with soil cations or adsorbed to various soil components (Singh and Satyanarayana, 2011). Phosphate diesters, referring to nucleic acids (such as DNA and RNA), phospholipids and teichoic acids, are more easily broken down and mineralized. 2) Phosphonates, characterized by the functional group C-P, are bound and present mainly in the form of 2-aminoethylphosphonic acid. They are less abundant and fairly stable in soil systems. 3) Phosphoric acid anhydrides, containing anhydride bonds and phosphate monoesters, play crucial roles in biochemical energy transfer in the form of ATP or NADPH.

Among these soil P fractions, bound inorganic P fractions are available for plants after solubilization, while organic P fractions need to be mineralized for uptake by plants (Spohn and Kuzyakov, 2013). The solubilization of Pi and the mineralization of Po are largely controlled by soil phosphorus solubilization microorganisms (PSMs) that secrete organic acids and synthesize various phosphatase enzymes (Richardson et al., 2009), including phosphorus solubilizing bacteria (PSB) and phosphorus solubilizing fungi (PSF). PSB account for 1% to 50% of the total soil microbial population, and are hence key actors controlling P availability, whereas PSF contribute only 0.1 - 0.5% to the P solubilization potential Khan et al. (2009). PSB converts insoluble inorganic phosphates to soluble H_2_PO_4_^-^ and HPO_4_^2-^ as they release protons and organic acids (e.g., gluconic acid, lactic acid and malic acid), which can chelate iron and aluminum cations to release Ca- or Fe-/Al- bound P (Bhattacharyya and Jha, 2012; Liu et al., 2014; Spohn et al., 2015). PSB can mineralize organic P to soluble inorganic P by producing extracellular phosphatase enzymes that catalyze the hydrolysis of esters and anhydrides of phosphoric acids (Nannipieri et al., 2011). There are three major types of phosphatase enzymes occurring in the soil system, including nonspecific phosphatases, phytases and phosphonatases (Rodríguez et al., 2006): 1) nonspecific phosphatases involved in dephosphorylation of phospho-esters or phosphoanhydrides from organic matter, 2) phytases that specifically release phosphoric acid from phytic acid (Singh and Satyanarayana, 2011), and 3) phosphonatases that break C-P from organophosphonates. The secretion of phosphatase enzymes is controlled by the expressions of corresponding functional genes in PSB, such as alkaline phosphatase (*phoD* and *phoA*), phytase (*appA*), and C-P lyases (*phn*).

The above contributions of PSB and PSF towards improving soil P availability, are complemented by soil arbuscular mycorrhizal fungi (AMF), which can form symbiotic associations with over 70% of plants (van der Heijden et al., 2015). These arbuscular mycorrhiza comprise an internal phase within the cortical cells of plant roots, and an external mycelium phase on plant root surfaces. The latter functions as an extension of the plant root system and transfers distant soil P to plants. Therefore, this mycorrhizal uptake pathway is a mechanism by which AMF promotes plant P uptake (Gosling et al., 2006; Lambers et al., 2008; Smith and Smith, 2011).

## 3 Potential mechanisms mediating the effects of soil chemical fumigation on soil phosphorus cycling

Soil P availability is largely determined by the rapid reactions of P anions with soil particles, as well as by the abundant soil microbial activity that converts orthophosphate into diverse organic forms or mineralizes organic P into soluble inorganic P forms (Achary et al., 2017). Soil properties, especially pH, determine the adsorption/desorption, and precipitation/dissolution processes of soil inorganic P. In addition, the soil microbiome plays a key role in the immobilization and mineralization processes affecting soil organic P (Jacoby et al., 2017).

Soil chemical fumigation, which introduces broad-spectrum biocides into the soil, can alter the soil microbiome. This affects soil P solubilizing microbial community structures, and consequently perturbs P cycling processes in the soil system. Although few studies have specifically investigated the effects of chemical fumigation on soil phosphorus, the effects of various fumigants on soil available P content have been reported in several studies on general soil health. These studies have mostly observed transient increases in soil available P content after soil chemical fumigation (Chen et al., 2020; Du et al., 2024; Huo et al., 2024). However, depending on the environmental conditions, crop type (Peng et al., 2022) and sampling time (Cheng et al., 2020), some studies have reported that chemical fumigants had decreased or had no significant effect on soil P availability.

**Figure 1:**
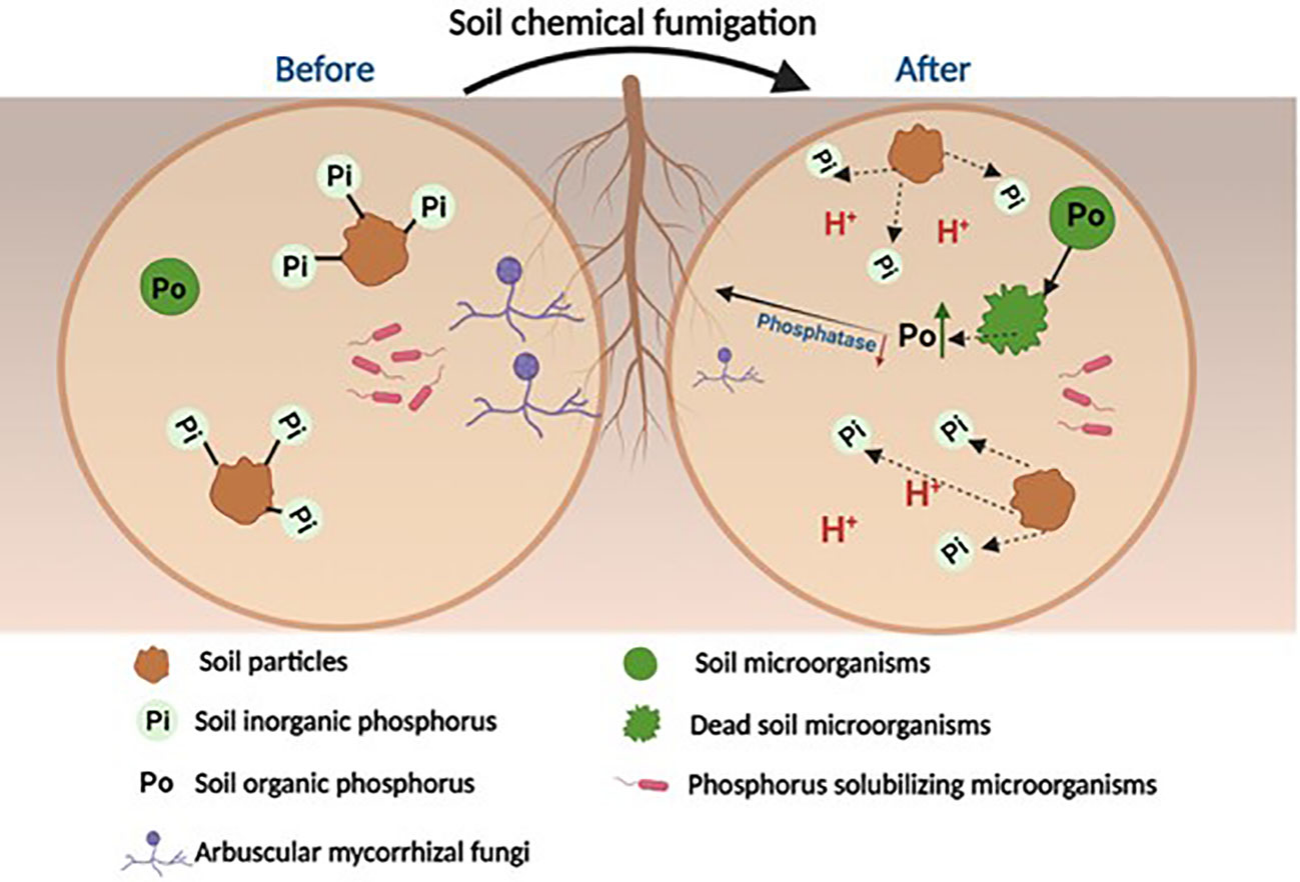
Conceptual diagram illustrating the soil phosphorus transformation processes that occur after chemical fumigation (Modified from Wang et al., 2024).

The precise molecular mechanisms of the biocidal effects induced by the various chemical fumigants remain uncertain, as past studies were mostly conducted as field or lab incubation experiments. Furthermore, these molecular mechanisms likely differ across the various chemical fumigants, which have different chemical properties and structures. Nevertheless, by analyzing the resulting changes to the soil microbiome and biogeochemical markers, it is possible to infer that chemical fumigants perturb soil P cycling processes through the following mechanisms (**Figure 1**). *Firstly, soil chemical fumigation may kill most soil microorganisms and release microbial P from dead microbial cells to the surrounding soil.* For example, Huang et al. (2020a; 2020b) found that soil fumigation using chloropicrin and dazomet increased soil active P (H_2_O-P, NaHCO_3_-Pi and NaHCO_3_-Po) in the early stage of the culture (<7days). Wang et al. (2023) also found that chloropicrin fumigation significantly increased the proportions of soil labile P fractions (Resin-P+NaHCO_3_-Pi + NaHCO_3_-Po) to total P across 28 weeks of ginger growth. *However, the death of PSMs may reduce P-cycle related functional bacterial genes, and slow down the secretion of related phosphatase enzymes, which may arrest the mineralization of soil organic P.* Both Huang et al. (2020a; 2020b) and Wang et al. (2023) found that soil fumigation significantly reduced the *phoC* and *phoD* gene copy number, and related acid and alkaline phosphatase activities. This in turn can further slow down the mineralization processes of organic P released from the dead microbes, which then results in an accumulation of soil organic P (especially labile NaHCO_3_-Po) in the fumigated soils (Wang et al. 2023). Similar decreases in soil phosphatase enzyme activities were also reported in several other studies (Klose & Tabatabai, 2002; Klose & Ajwa, 2004). However, there is a lack of research on the chemical structures of the compounds comprising soil organic P, which could provide direct evidence on the molecular mechanisms that link changes in soil phosphatase enzymes to the transformation of soil organic P.

*In addition, cell lysis may also result in the release of various organic acids, which will lower the soil pH and promote the solubilization of inorganic P fractions (* *Wang et al., 2022b* *).* These secreted organic acids may replace bound-P by forming complexes with Fe/Al/Ca-oxyhydroxides and releasing Pi into the soil solution (Lopez-Arredondo et al., 2014). Several previous studies, such as Wang et al. (2023) and Li Q. et al. (2022), have found that soil fumigation significantly reduces soil pH values, which provides indirect evidence for this hypothesis. However, some other studies such as Cheng et al. (2021) and Zhang et al. (2019a) have found that soil fumigation significantly increases soil pH values, possibly explained by soil fumigation significantly inhibiting soil nitrification processes (Yan et al., 2017). To resolve this uncertainty, it is necessary to study in greater detail the changes to soil organic acids in fumigated soils, in particular the chemical species and the amount involved.

Overall, the existing literature allows us to conclude that soil chemical fumigation could increase the amount of soil available P in the short term. However, soil fumigation is not a sustainable method for improving soil P availability for uptake by plants. *In addition to PSB, soil fumigation may also eliminate soil AMF, which will disrupt plant P uptake through the mycorrhizal uptake pathway*. For example, Dangi et al. (2017) found that soil fumigation significantly decreased the relative abundance of AMF. Wang et al. (2022a) found that although the total P uptake by ginger plants doubled under chloropicrin fumigation compared to untreated soils, the ginger physiological P use efficiency was halved. The responsible mechanism in this case was that the soil available P released following soil fumigation was used mostly for ginger shoot growth at early growth stages, exacerbating the soil P deficiency that affected ginger rhizome growth at later stages. Therefore, the increase in soil available P content and the decrease in AMF activity following fumigation may cause transient, and ultimately uncertain, effects on P uptake by plants.

In addition, as the soil is fumigated an increasing number of times, poorly resistant microorganisms may gradually disappear, and microorganisms with a stronger resistance to fumigation will increasingly dominate the soil microbiota, possibly leading to irreversible changes to the soil microbiome (Dangi et al., 2017; Li et al., 2017; Zhang et al., 2020; Zhou et al., 2020). Therefore, decreases in the related PSMs and phosphatase enzyme activities may lead to serious detrimental effects on soil P availability in the long run, if the soil is repeatedly fumigated to the point where the soil microbiome is irreversibly damaged. For example, Dangi et al. (2017) concluded that there were significant differences in soil microbial communities between untreated sites and sites with 15 or 30 year histories of annual fumigation with methyl bromide. AMF colonization was significantly reduced in all fumigated sites. Wang et al. (2022b) observed that the soil labile P fraction was significantly lower in ginger fields subjected to three consecutive years of chloropicrin fumigation, compared to new ginger fields fumigated once. Therefore, short-term and long-term fumigation may lead to different effects on soil P availability, each associated with different mechanisms characterized by different time scales.

In addition to the changes in the soil P availability, the effects of soil fumigation on plant P uptake also depends on crop type. Usually, soil P content increases immediately after soil chemical fumigation, which means that soil chemical fumigation might have a more significant positive effect on soil P availability in the early crop growth period. For crops harvested in early growth stages, such as leaf vegetables, soil P availability can be improved by soil chemical fumigation, and the application of P fertilizers could be reduced. However, for crops harvested at the later growth stages, such as root vegetables, soil P availability might be greatly reduced due to a decrease in soil phosphatase enzyme activity and related PSMs. Here, P fertilizer application should be increased at later crop growth stages to meet the needs of the root system development. Some studies have proposed altering P fertilization practices and timings to be synergistic with soil fumigation. Huang et al. (2020a) suggested that P fertilizer loads should be reduced at the early stages of crop growth immediately following fumigation (< 30 days). Wang et al. (2023) suggested that reducing early P fertilization aggravates possible P deficiencies arising at later stages of crop growth, and therefore that additional P fertilization should be applied then.

*In addition to soil P cycling, soil chemical fumigation can also alter soil carbon and nitrogen cycling processes (* *Fang et al., 2022* *), ultimately altering the C:N:P stoichiometry in soils*. Soil microorganisms flourish best under a stable and optimal C:N:P stoichiometry (Dai et al., 2020). Fluctuating or unbalanced C:N:P stoichiometries may thus influence the growth of PSMs, and the mineralization processes of organic P (Heuck et al., 2015). Therefore, when studying P fertilization practices in fumigated soils, other soil nutrient cycles (such as C, N, K), and interactions between these cycles (particularly N–P interactions) (Vitousek et al., 2010; Fageria & Oliveira, 2014; Hu & Chu, 2020), should also be considered.

## 4 Drawing a distinction between the effects of fumigants and fumigation

The literature on soil fumigation typically does not distinguish between the effects of the fumigants, and those of other factors involved in the soil chemical fumigation process. Soil P availability and its dynamics are complex, and should be seen as the overall combined effects of the entire process of soil fumigation on soil physical, chemical and biological properties, and not solely as the result of the biocidal effects of the fumigant. During soil chemical fumigation, plastic mulch is often applied as a tarp to trap the gaseous fumigants in the soil system to increase effectiveness (Austerweil et al., 2006; Fang et al., 2017; Qin et al., 2017; Wang et al., 2019), and prevent emissions into the surrounding atmosphere where fumigants pose health and ecotoxicity risks (Yates et al., 2002, 2011). Plastic tarps are usually installed immediately after fumigant application and removed no less than 2 weeks later (Gao et al., 2013). The effectiveness of P fertilization in increasing P availability may be adversely affected by plastic mulching (Gu et al., 2018). Several years of continuous plastic-film mulching may result in an increase to the proportions of soil water-stable macroaggregates (>0.25mm) (Wang et al., 2017), which may cause more particulate P to be occluded during macroaggregate formation and therefore become unavailable for uptake by plants (Garland et al., 2018). Mulching also alters soil temperature, aeration, moisture, and microorganism ecology (Khan et al., 2000; Sinkevičienė et al., 2009), which then affects the transport, diffusion, cycling, and availability of P (Watanabe et al., 1960; Power et al., 1964; Turner & Gilliam, 1976; Wu et al., 2022). Hence, the effects of the chemical fumigation practice on the soil microbial community are likely not solely due to biogeochemical interactions with the fumigant, but also the physicochemical effects of mulching. Furthermore, as mulching facilitates soil moisture retention, mulched fields are often irrigated less intensively and less frequently (Kumar & Dey, 2011; Chukalla et al., 2015; Salem et al., 2021), which in turn may reduce P diffusion and availability (Kargbo et al., 1991).

Recent research suggests that plastic residues left in the soil may also directly influence nutrient adsorption and transport, soil biogeochemistry, and nutrient cycling processes (Gao et al., 2019; Guo et al., 2023; Yin et al., 2023; Ding et al., 2024). In turn, the effects of the plastic residues depend on tillage practices (Serrano-Ruiz et al., 2021; Calero et al., 2023), the history of mulch and fertilizer application (Zhang Z. et al., 2022; Guo et al., 2024), and the age of the residual microplastics (Ju et al., 2024).

The confounding effects of plastic mulching will likely be amplified by the growing interest in reusing old (i.e. weathered) plastic mulches (Zhang X. L. et al., 2022) and biodegradable plastic mulches (DeVetter & Stanghellini, 2020; Madrid et al., 2022). Biodegradable and weathered plastics may cause larger disruptions to soil microbial ecology and biogeochemistry as they may more readily react chemically with the fumigants (DeVetter & Stanghellini, 2020; Campanale et al., 2023; Ju et al., 2024), and can be metabolized more readily by soil organisms (Serrano-Ruiz et al., 2021; Yu et al., 2023). Hence, aged and biodegradable plastics are more likely to substantially alter soil microbial communities, and cause larger and more rapid onset of negative effects on crop germination and growth (Li et al., 2021; Ju et al., 2024).

## 5 Knowledge gaps to be addressed in future research

This review highlights the side effects of soil fumigation on soil P availability, discusses potential mechanisms underlying these processes, and provides recommendations for post-fumigation P fertilization practices. In order to design environmentally sustainable agricultural practices to maximize crop yields, knowledge gaps in the following topics have to be addressed more thoroughly:

1) We have some understanding of the changes to soil P availability that results from soil chemical fumigation, but the molecular mechanisms that alter soil P availability remain unclear, though changes to the microbiome appear to be a significant contributor. We recommend investigating how to utilize keystone microbe species to improve soil P availability and prevent outbreaks of crop diseases, and to exploit interactions between the cycles of various nutrients to control soil P availability. Furthermore, the soil microbiome and its response to soil fumigation may be location-specific, thus all generalized conclusions should be verified and optimized within the local context. Further studies are also needed on how to adjust P fertilizer application in response, especially taking into account nutrient stoichiometry, to improve the soil nutrient use efficiency and avoid potential resource wastage and environmental pollution.

2) Soil-borne diseases may alter the uptake, translocation, distribution and utilization of nutrients by crops. Conversely, changes in soil nutrient availability induced by fertilization may also affect plant disease severity by altering the abundance of soil-borne pathogens or plant disease resistance. The effects of fertilization on plant disease susceptibility and pathogen ecology vary with plant species, and the rhizosphere–microbiome interactions characteristic of each plant species. Further research should be conducted to develop specific fertilization protocols for each crop type.

3) As the use of plastic mulch to trap fumigants in the soil is often a key step in the fumigation process, findings on the effects of fumigants on soil biogeochemistry and agricultural productivity are essentially confounded with the effects of plastic mulch application and residues. Future research into the effects of soil fumigation on P cycling should control for the effects of the applied plastic mulch, and the residual microplastics they leave behind, especially as these residues accumulate, degrade, and disintegrate in the soil in the long term (Wahl et al., 2024; Li S. et al., 2022; Liu et al., 2022; Briassoulis, 2023). This is to distinguish the effects caused by the mulching process, mulch residues, the fumigant itself, and interaction effects between the fumigant and mulch (e.g. the permeability of the fumigant through the mulch, net effects on soil microbial ecology, and chemical interactions between the fumigant and mulch). If plastic mulching is implicated in adverse effects to agricultural productivity, other forms of surface sealing (e.g. water sealing, surface chemical treatment) (Gao et al., 2011) may be evaluated for the soil chemical fumigation process.

Altogether, the effects of various fumigants on soil P availability vary with environmental conditions, soil type, agricultural practices, and monitoring duration, as the effects may be transient and dynamic. Meta-analysis research may be necessary in order to sufficiently and comprehensively address the effects of the various contributing factors, and to make generalizable conclusions on the complex and dynamic effects of fumigants on soil P availability.

## Author contributions

YW: Writing – original draft. DT: Writing – original draft.
